# Case Report on Renal Failure Reversal in Lambda Chain Multiple Myeloma with Bortezomib and Dexamethasone

**DOI:** 10.1155/2014/940171

**Published:** 2014-06-19

**Authors:** Bhanu K. Patibandla, Akshita Narra, Ahmad A. Alwassia, Anthony Bartley, Gurprataap S. Sandhu, James Rooney, Robert M. Black

**Affiliations:** ^1^Department of Medicine, Saint Vincent Hospital, University of Massachusetts School of Medicine, 123 Summer Street, Worcester, MA 01608, USA; ^2^Department of Medicine, University of Connecticut, Farmington, CT 06030, USA; ^3^Department of Medicine, University of Pittsburgh Medical Centre, 200 Lothrop Street, Pittsburgh, PA 15206, USA

## Abstract

Renal failure (RF) reversal in multiple myeloma (MM) is associated with an improved prognosis. Light chain myeloma, serum creatinine (SCr) > 4 mg/dL, extensive proteinuria, early infections, and certain renal biopsy findings are associated with lower rates of RF reversal. Our patient is a 67-year-old female with multiple poor prognostic factors for RF reversal who demonstrated a rapid renal response with bortezomib and dexamethasone (BD) regimen. She presented initially with altered mental status. On exam, she appeared lethargic and dehydrated and had generalized tenderness. She had been taking ibuprofen as needed for pain for a few weeks. Labs showed a white cell count—18,900/*μ*L with no bandemia, hemoglobin 10.8 gm/dL, potassium—6.7 mEq/L, bicarbonate—15 mEq/L, blood urea nitrogen—62 mg/dL, SCr—5.6 mg/dL (baseline: 1.10), and corrected calcium—11.8 mg/dL. A rapid flu test was positive. Imaging studies were unremarkable. Her EKG showed sinus tachycardia and her urinalysis was unremarkable. The unexplained RF in an elderly individual in conjunction with hypercalcemia and anemia prompted a MM work-up; eventually, lambda variant MM was diagnosed. An immediate (4 days) renal response defined as 50% reduction in SCr was noticed after initiation of the BD regimen.

## 1. Introduction

Renal failure (RF) is common in multiple myeloma (MM) [1] and is associated with a poor prognosis [2, 3]. The pathology of RF in MM is heterogeneous [4]; it is most often associated with immunoglobulins, especially free light chain (FLC) deposition [5–8]. FLC can cause broad spectrum of renal lesions [9, 10]; myeloma cast nephropathy (MCN) is the most common [5, 11]. FLC damages kidneys due to its direct toxic effect on proximal convoluted tubules (PCT) and subsequently triggers inflammatory pathways and cast formation [6]. Excess FLC production in MM beyond the endocytosis capacity of the PCT initiates this renodestructive cascade [12]. Of note, the extent of renal disease does not correlate with quantity of the FLC. This variable toxicity can be attributable to the functional and morphological differences of the FLC subtypes—Kappa (*κ*)/Lambda (*λ*) [13–15] apart from the status and influence of the concomitant comorbidities in MM, for example, dehydration, hypercalcemia [16], and infections [7, 8, 17, 18].

Reversal of RF in MM is associated with improved survival [2]; FLC myeloma, serum creatinine (SCr) > 4 mg/dL, extensive proteinuria, early infections, and certain renal biopsy findings (interstitial fibrosis and more tubular casts) are associated with lower rates of RF reversal [19–21]. Interestingly, female gender was also associated with poor RF recovery in Rota et al.'s study [22], but this association was not consistent in other studies.

With the advent of novel agents, such as thalidomide and bortezomib with/without high-dose dexamethasone, RF reversal rates have improved [23–26]. Further studies on novel agents reported bortezomib plus high-dose dexamethasone (BD) regimen to be more efficacious and possibly having renal protective effect [27–35]; therefore, this combination has become preferred therapy in MM with RF [36]. However, poor prognostic factors for RF reversal persisted even with these newer agents [24, 34].

We present a MM case with multiple poor prognostic factors for RF reversal (i.e., *λ* LC MM, severe acute RF with SCr of >4 mg/dL, eGFR < 15 mL/min, and female gender) demonstrating a rapid renal response with the BD regimen. A renal response was defined as 50% reduction in SCr; compared to the pretreatment values [25, 37], the renal response time in our case was short (4 days) and was achieved with chemotherapy alone without adjunctive plasmapheresis or dialysis.

## 2. Case

A 67-year-old female presented with an acute change in mental status manifested as confusion, transient aphasia, and brief phase of unresponsiveness. She had generalized malaise, body aches, and fatigue of few days' duration. She had been taking ibuprofen tablets—200 mg every 4–6 hours as needed for pain since few weeks. She was hospitalized ten days prior for viral gastroenteritis (GE); then, she received contrast for CT abdomen and pelvis (A & P) which ruled-out intra-abdominal pathology.

Past medical history included well-controlled treated hypertension (HTN), chronic kidney disease (CKD) stage III (baseline SCr 1.10 mg/dL), anemia of chronic disease (ACD), chronic back pain secondary to lumbar disk degenerative disease, and polymyalgia rheumatica (PMR). Home medications included Lisinopril, Multivitamins, and Prednisone (2 mg twice daily for PMR). Family history is negative for any autoimmune disorders or MM. She is a reformed smoker, social drinker, and denied illicit drug use ever.

Physical examination revealed a Caucasian female who was lethargic but responsive to verbal stimuli, oriented to person and not to place and time. Other significant findings included dry oral mucosa, generalized abdominal, and extremity tenderness. She was afebrile and the rest of the vitals included blood pressure—84/53 mm Hg, pulse—104 beats/minute and regular, respiratory rate—16/minute, and pulse oximetry—96% on room air.

Laboratory investigations at the time of admission included Complete Blood Count (CBC), Basic Metabolic Panel (BMP), hepatic panel, urinalysis, blood cultures, and few others. CBC showed white blood cell (WBC) count—18,900/*μ*L with no bandemia, hemoglobin—10.8 gm/dL, and platelets—211,000/*μ*L. BMP showed sodium (Na)—132 mEq/L, potassium—6.7 mEq/L, chloride—100 mEq/L, bicarbonate—15 mEq/L, blood urea nitrogen (BUN)—62 mg/dL, SCr—5.60 mg/dL, glucose—96 mg/dL, calcium—11.2 mg/dL, magnesium—1.8 mg/dL, and phosphorous of 4.5 mg/dL. Lactate was 1.0 mg/dL. Hepatic panel was unremarkable other than mild hypoalbuminemia of 3.2 mg/dL, thus making corrected calcium of 11.8 mg/dL. Urine (U) dip-stick showed small protein (1+), and blood; U microscopy was negative for any kind of casts. U-Na—100 mEq/L, U-Cr—29 mg/dL, U-specific gravity (U-SG)—1.007, U-protein—321.0 mg/dL, and U-albumin—58.4 mg/dL; therefore, U-albumin excretion (UAE) was 2.01, and fractional excretion of Na (FENa) was 14.6%. Estimated GFR (eGFR) was 7.0 mL/min. Chest X-ray (CXR) did not show any cardiopulmonary disease. CT brain ruled out stroke. Noncontrast CT A & P did not show any acute intra-abdominal pathology; there was no evidence of hydronephrosis, masses, or lymphadenopathy. EKG showed sinus tachycardia without any changes specific to hyperkalemia. Rapid influenza test was positive for type B virus.

**Figure 1:**
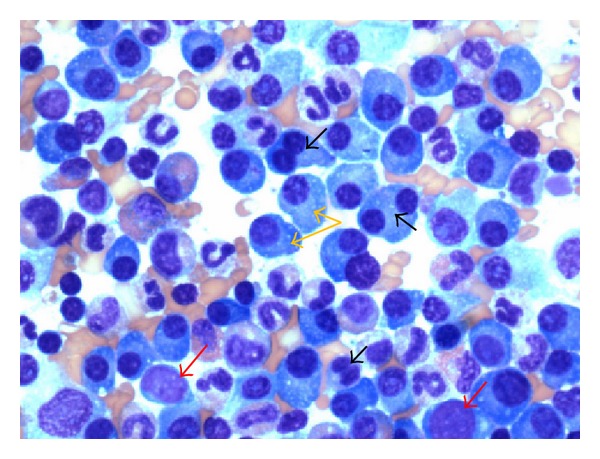
Wright-Giemsa stain of the bone marrow aspirate demonstrating more than 50% of morphologically variable plasma cells including binucleate forms (black arrows), mature plasma cells with large basophilic cytoplasm, eccentric nucleus with perinuclear halo and clock face chromatin (orange arrows), and immature cells with high nuclear-cytoplasm ratio and dispersed chromatin (red arrows).

**Figure 2:**
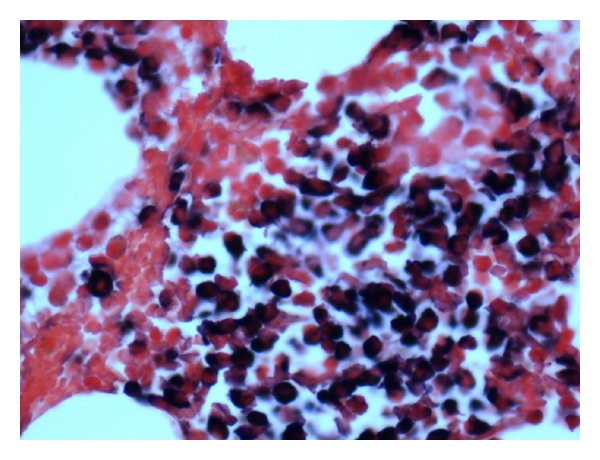
Immunohistochemistry of the bone marrow biopsy showing positivity for lambda light chains.

She was admitted with working diagnoses of severe sepsis secondary to influenza, acute RF, hyperkalemia, and hypercalcemia. She was started on oseltamivir treatment (75 mg oral twice daily for 10 days) for severe influenza. Intravenous fluids were initiated; bicarbonate, dextrose plus insulin, and sodium polystyrene were given for hyperkalemia. Over the initial couple of days, her mental status and hemodynamics improved; leukocytosis and electrolyte abnormalities got normalized. Her urine output has been adequate. However, RF continued to worsen despite fluid resuscitation, correction of hypercalcemia, and avoidance of nephrotoxins. She did not have any clinical indications that would have warranted dialysis. In view of this unexplained acute RF in an elderly female with concomitant findings of hypercalcemia and chronic anemia, we considered the possibility of MM and ordered serum protein electrophoresis (SPEP). SPEP revealed monoclonal M spike in the beta region. Serum M protein concentration was 0.5 gm/dL and all immunoglobulin levels were low suggestive of hypogammaglobulinemia. Serum and urine immunofixation (IFE) were positive for monoclonal gammopathy in lambda region. Serum FLC analysis showed *κ* LC levels at 4.56 mg/L (normal range: 3.30–19.40 mg/L) and *λ* LC levels 21,480 mg/L (5.70–27.60 mg/L) with *λ*/*κ* ratio of 4710. FLC levels were confirmed with dilution. Bone marrow biopsy was performed; Wright Giemsa staining of the bone marrow aspirate demonstrated more than 50% of morphologically variable plasma cells characteristic of MM (Figure 1), and immunohistochemistry confirmed lambda LC restricted plasma cells (Figure 2). Bone survey with plain radiographs for prognostic evaluation and risk stratification was considered, but the patient declined. Imaging studies done in the past were reviewed which included CT brain, A & P, and CXR; chronic bilateral sacral fractures and osteopenia with cystic changes in femoral head were observed; there were no lytic lesions characteristic of MM.

**Figure 3:**
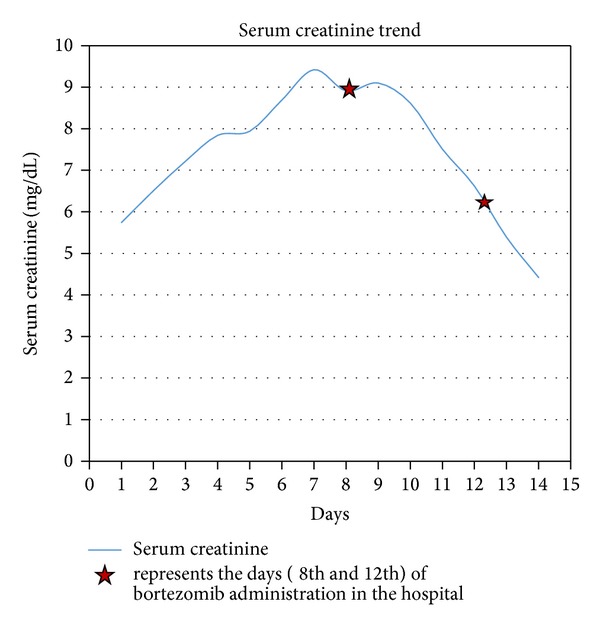
Serum creatinine trend over the course of the hospitalization.

Chemotherapy with bortezomib (1.3 mg/m^2^ subcutaneous injection on days 1, 4, 8, and 11 q. 21 days schedule) and high dose dexamethasone (20 mg per oral daily for 5 days a week) was initiated promptly after the diagnosis of MM. There was a 50% reduction in SCr and SFLC concentration after two doses of bortezomib and five days of dexamethasone (Figure 3). She was discharged home with an outpatient chemotherapy arrangement. Her renal function improved with SCr to 2.5 mg/dL and eGFR to 18 mL/min after two chemotherapy cycles and stabilized thereafter while lambda light chains were still elevated at 11,300 mg/L.

## 3. Discussion

The initial differential diagnoses considered for an acute RF in our case included (i) prerenal—due to hypovolemia secondary to viral GE and sepsis induced hypotension; and (ii) intrinsic renal—acute tubular necrosis (ATN) due to prolonged hypovolemia and/or nephrotoxins (NSAIDs and CT contrast), or allergic interstitial nephritis (AIN). CT A & P done at the index hospital admission ruled out obstructive uropathy. Preliminary work-up including the urine studies did not support a prerenal etiology (i.e., BUN/SCr ratio~11 : 1, U-Na: 100 mEq/L, FENa~14%, and U-SG: 1.007) and the bland urine sediment was less likely to suggest ATN or AIN. MM was considered in this setting of an unexplained RF in an elderly individual [8, 38]; furthermore, the concomitant findings of hypercalcemia, ACD, and bone pains, as in our case, improves diagnostic sensitivity for MM, which prompted us to do relevant investigations (SPEP, IFE, and bone marrow biopsy) eventually diagnosing MM.

Of note, her SCr was 1.01 mg/dL during the previous hospitalization with viral GE, 10 days prior to the index hospitalization. On this admission, the LC concentration in the renal tubules might have reached a toxic level, the effect of which could have been further precipitated by hypotension due to viral GE/sepsis, hypercalcemia, and nephrotoxins (NSAID's and CT contrast). While RF in our patient could be multifactorial due to hypotension, hypercalcemia, and nephrotoxins, it is important to note that there was worsening of the renal function despite correction of the above mentioned factors. Furthermore, the prompt renal response noticed to chemotherapy favors MM as the principle underlying etiology of acute RF. We deferred renal biopsy as the clinical presentation and laboratory investigations (*λ* LC MM, low UAE [39], and bland urine sediment) in our case were highly suggestive of MCN. Moreover, it was felt that the procedural risks of the biopsy would outweigh the limited prognostic information it would confer.

The main strategy in the management of RF in MM is to lower FLC concentration immediately. FLC concentration can be lowered by slowing their production through chemotherapy, mechanical removal through dialysis/plasmapheresis, and diluting FLC in renal tubules through hydration and removal of factors precipitating cast formation. Previously, mechanical removal of FLC was the most often sorted treatment strategy [40, 41]; furthermore, lack of effective antimyeloma chemoagents hindered renal recovery, thus leading to greater dialysis dependency and deaths in the past [39]. With the advent of rapid acting novel agents showing improved renal recovery, myeloma response, and over-all prognosis, prompt chemoinitiation with bortezomib-based regimen has become the standard strategy [42]. Sometimes, patients might have been already initiated on hemodialysis for advanced RF with a pending or established diagnosis of MM; in this dialysis-dependent population as well, bortezomib was shown to be well tolerated [43, 44] compared to other conventional chemoagents [45, 46] and, furthermore, novel agents in combination with high-dose dexamethasone improved renal function rapidly to become independent of dialysis sooner [23, 44].

Most commonly, renal recovery correlates with reduction in SFLC load as noticed in our case; this association has been particularly reported in newly diagnosed MM [37] and biopsy proven MCN [47]—Leung et al. reported that renal response was not noticed unless there was a 50% or more reduction in SFLC load and suggested to consider plasmapheresis as a bridge or adjunctive therapy in biopsy proven MCN cases not responding immediately to chemotherapy as the chronicity of RF can affect the degree of recovery. Otherwise, there are no standard recommendations on when to resort to plasmapheresis in case of MM nonresponsive to chemotherapy. Few studies evaluated combination strategies, that is, mechanical approaches in adjunct to novel agents [48, 49] in lowering SFLC load. But, they were limited in providing information on the relative contribution of mechanical removal and, furthermore, early reduction in SFLC load probably reflects the efficacy of the chemotherapy [50]; rapid reduction in SFLC load in our case with just BD regimen alone supports the later.

In conclusion, our case showed that bortezomib and dexamethasone regimen alone may be effective in renal recovery even in patients with severe RF in MM. Future studies comparing outcomes between combination strategies and novel agents alone are warranted to understand the additional benefits with mechanical modalities and, importantly, when the combination strategy would be needed—EuLITE [51] and MYRE [52] trails are in progress in this regard.
